# Inter-hospital transfer of polytrauma and severe traumatic brain injury patients: Retrospective nationwide cohort study using data from the Swiss Trauma Register

**DOI:** 10.1371/journal.pone.0253504

**Published:** 2021-06-18

**Authors:** Rebecca M. Hasler, Thomas Rauer, Hans-Christoph Pape, Marcel Zwahlen

**Affiliations:** 1 Department of Traumatology, University Hospital Zürich, Zürich, Switzerland; 2 Institute of Social and Preventive Medicine (ISPM), Bern University, Bern, Switzerland; Assiut University Faculty of Medicine, EGYPT

## Abstract

**Introduction:**

Polytrauma and traumatic brain injury (TBI) patients are among the most vulnerable patients in trauma care and exhibit increased morbidity and mortality. Timely care is essential for their outcome. Severe TBI with initially high scores on the Glasgow Coma (GCS) scores is difficult to recognise on scene and referral to a Major Trauma Center (MTC) might be delayed. Therefore, we examined current referral practice, injury patterns and mortality in these patients.

**Materials and methods:**

Retrospective, nationwide cohort study with Swiss Trauma Register (STR) data between 01/012015 and 31/12/2018. STR includes patients ≥16 years with an Injury Severity Score (ISS) >15 and/or an Abbreviated Injury Scale (AIS) for head >2. We performed Cox proportional hazard models with injury type as the primary outcome and mortality as the dependent variable. Secondary outcomes were inter-hospital transfer and age.

**Results:**

9,595 patients were included. Mortality was 12%. 2,800 patients suffered from isolated TBI. 69% were men. Median age was 61 years and median ISS 21. Two thirds of TBI patients had a GCS of 13–15 on admission to the Emergency Department (ED). 26% of patients were secondarily transferred to an MTC. Patients with isolated TBI and those aged ≥65 years were transferred more often. Crude analysis showed a significantly elevated hazard for death of 1.48 (95%CI 1.28–1.70) for polytrauma patients with severe TBI and a hazard ratio (HR) of 1.82 (95%CI 1.58–2.09) for isolated severe TBI, compared to polytrauma patients without TBI. Patients directly admitted to the MTC had a significantly elevated HR for death of 1.63 (95%CI 1.40–1.89), compared to those with secondary transfer.

**Conclusions:**

A high initial GCS does not exclude the presence of severe TBI and triage to an MTC should be seriously considered for elderly TBI patients.

## Introduction

Despite the enormous progress in the treatment of severely multiply-injured patients worldwide, trauma is still a major cause of premature death and permanent disability [1–3]. Although the implementation of standardized guidelines for preclinical care and the ongoing optimization of the organization of trauma networks have led to improved outcomes for trauma patients worldwide, the preclinical care of multiple trauma patients remains challenging for the emergency medical service [1, 4–7]. One of the greatest of these challenges and the key to the effectiveness of a trauma system is the appropriate prehospital triage to transport the patients as quickly and safely as possible to the nearest and most suitable hospital, in accordance with the philosophy of getting the “right patient to the right place at the right time” [1, 2, 8, 9]. Due to the limited diagnostic possibilities and resources in the prehospital environment, the triage may be incorrect and lead to undertriage or overtriage [1, 3, 10–13]. Undertriage is defined as a decision not to refer patients with severe injuries to a high-level trauma center [2, 3]. Overtriage is defined as a decision to refer patients without severe injuries to a high-level trauma center [2, 3]. There is some evidence that geriatric patients are often undertriaged [1, 11, 12, 14–16]. While it is generally agreed that overtriage results in an unnecessary burden on high-level trauma centers [3, 13, 17, 18], there is some controversy as to whether undertriage and subsequent interfacility transfers to a high-level trauma center may lead to increased mortality and morbidity [3, 9, 19, 20] or whether this type of transport has little or no impact on mortality compared to direct transport to a high-level trauma center [21, 22]. Inefficient triage and subsequent inter-hospital transfer lead to increased costs, due to repeated diagnostic investigations and an extended length of stay in the emergency department (ED) [1, 23, 24]. Consequently, there is a need for better allocation of medical resources in polytrauma by optimizing the rates of both over- and undertriage [2]. Therefore the Center for Disease Control and the American College of Surgeons recommends a target undertriage rate of less than 5%, while the overtriage rate should not exceed 25–35% [2, 3, 10, 25]. No current data are available on prehospital triage of polytraumaand traumatic brain injury (TBI) patients in Switzerland. The aim of this study is, for the first time, to use data of the Swiss Trauma Register (STR) to shed light on inter-hospital transfers and mortality of severely multiply-injured and TBI patients in Switzerland. Our hypotheses are that mortality varies according to injury type (patients with and without [isolated] TBI) and that age as well as the presence of TBI are independent factors for inter-hospital transfer.

## Materials and methods

### Study type

We conducted a retrospective cohort study using data from the STR for the period from 01/01/2015 to 31/12/2018. STR data collection started in 2015 and includes adult (≥16 years) patients with an Injury Severity Score (ISS) >15 or an Abbreviated Injury Scale (AIS) for head trauma >2, admitted to one of the 12 Major Trauma Centres (MTC). The primary outcome was mortality according to injury type [26]. All data were fully anonymized before we accessed them and the ethics committee waived the requirement for informed consent.

### Data preparation

**Fig 1:**
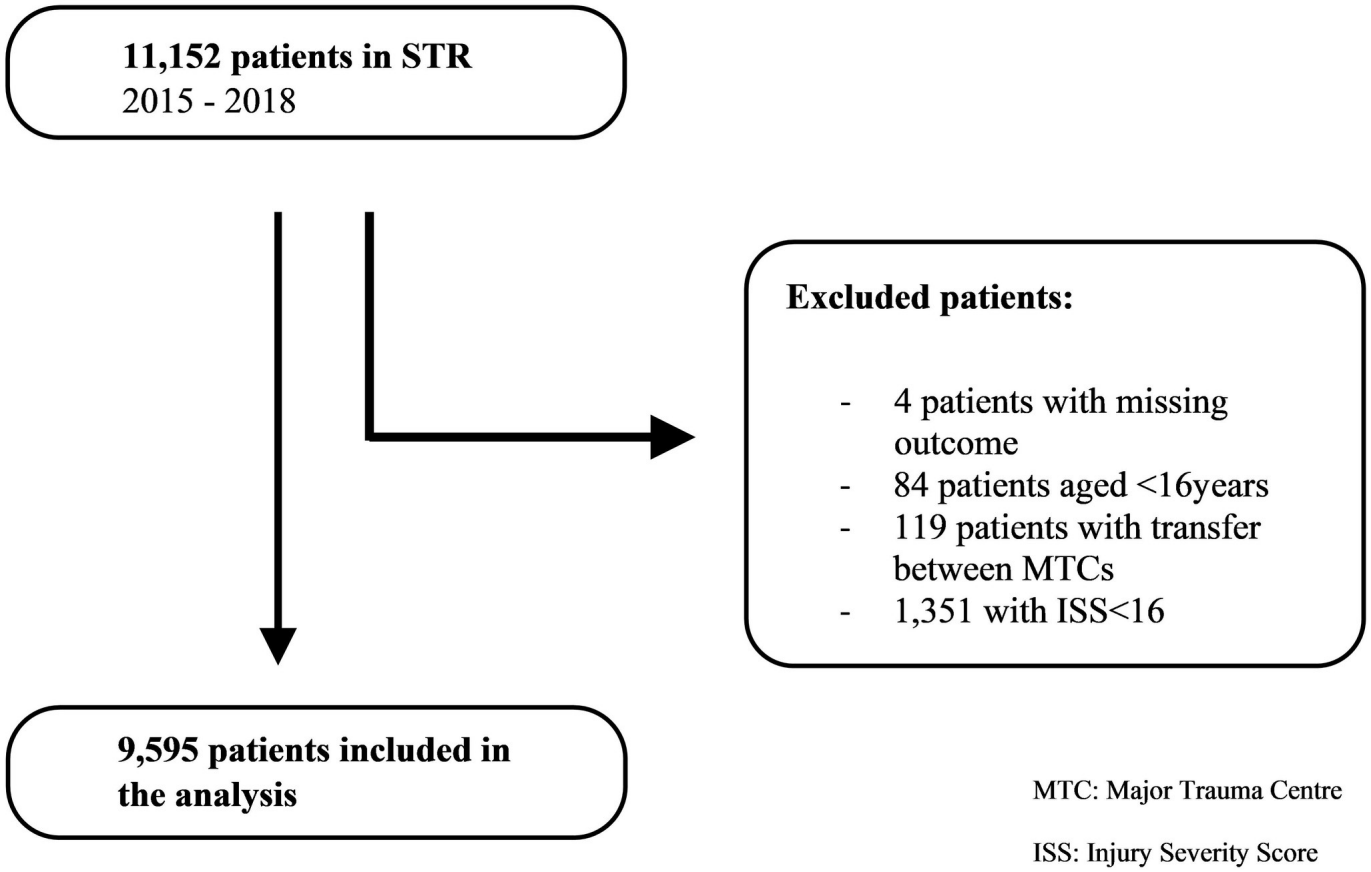
Patients`flow chart.

STR allows its participating hospitals to submit patients not meeting the official inclusion criteria for registry and data collection purposes. Therefore, we excluded them from the data set before analysis (Fig 1).

We grouped age into intervals starting from 16–24 years and ending with ≥85 years. The age groups built comprise ten years after the first group that comprises 8 years. We differentiated patients directly admitted to an MTC and patients secondarily transferred from a regional hospital to an MTC. We excluded patients with transfer between MTCs, patients with missing data for death or discharge and patients aged <16 years.

We used the AIS head>2 codes of the STR to define patients with severe traumatic brain injury (TBI) (S1 Appendix). For the analysis, we created 3 groups of injured patients: 1) polytrauma patients with an ISS>15 and without severe TBI; 2) polytrauma patients with an Injury Severity Score (ISS) >15 and concomitant severe TBI; and 3) patients with isolated severe TBI.

We used the Glasgow Coma Scale (GCS) Score determined on admission to the ED and grouped it into a) 3; b) 4–5; c) 6–8; d) 9–12 and e) 13–15 points, as published previously [27–29].

The ISS was used as a continuous variable in the analyses.

### Data analysis

Firstly, we displayed the patients`characteristics using descriptive statistics (numbers, percentages, mean/median). Observation time for survival analysis, with death as outcome, started at hospital entry and ended at date of death or discharge, whichever came first. We present results from Cox proportional hazard models for injury type as the independent variable. Further analyses were adjusted for age group, gender, GCS, MTC, transfer type and ISS. *We included patients with missing GCS data as “missing” as a separate category in the Cox model in order to avoid introducing selection bias by excluding these patients from the analysis*. Additionally, we checked for interactions between transfer type and age groups by incorporating appropriately defined interaction terms and testing for the whole group of interaction terms. Results are presented accordingly, with separate analyses by injury type. A p value of <5% was taken as statistically significant. All analyses were completed using STATA, Release 15.0 (StataCorp LP, College Station, TX).

### Ethical approval

We received ethical approval from the cantonal ethics committee of Zürich, BASEC No Req-2020-00592.

## Results

### Study population

Between 1^st^ January 2015 and 31^st^ December 2018, 11,152 patients were entered into the STR database. After excluding patients with missing data on death or discharge and patients not matching the inclusion criteria, 9,595 patients were analysed (Fig 1); 69% (n = 6,584) were men. Median age was 61 years and median ISS 21. Overall mortality was 12% (n = 1,194). Two thirds of patients (69%, n = 6,599) had a favourable GCS—between 13 and 15 on admission to the ED (Table 1).

**Table 1:** Baseline characteristics of study patients.

Variable	Survived (%)	Died (%)	Total
**Age group**			
16–24	793 (94.0)	51 (6.0)	844
25–34	897 (93.7)	60 (6.3)	957
35–44	816 (93.9)	53 (6.1)	869
45–54	1,201 (93.4)	84 (6.5)	1,285
55–64	1,276 (91.3)	122 (8.7)	1,398
65–74	1,239 (86.1)	200 (13.9)	1,439
75–84	1,367 (80.3)	336 (19.7)	1,703
≥85	812 (73.8)	288 (26.2)	1,100
**Gender**			
Male	5,833 (88.6)	751 (11.4)	6584
Female	2,568 (85.3)	443 (14.7)	3011
**Glasgow Coma Scale**			
3	886 (57.3)	660 (42.7)	1,546
4–5	39 (53.4)	34 (46.6)	73
6–8	196 (75.7)	63 (24.3)	259
9–12	602 (81.4)	138 (18.7)	740
13–15	6,369 (96.5)	230 (3.50)	6,599
missing	309 (81.8)	69 (18.3)	378
**Secondary Transfer**			
Yes	2,289 (91.9)	203 (8.1)	2,492
No	6,112 (86.0)	991 (14.0)	7,103
**Injury Severity Score**			
(Median)	20	26	21
**TBI**^**2**^			
Yes	4,662 (85.7)	779 (14.3)	5,441
No	3,739 (90.0)	415 (10.0)	4,154
**Isolated TBI**			
Yes	2,392 (85.4)	408 (14.6)	2,800
No	6,009 (88.4)	821 (11.6)	6,795

1) Severe traumatic brain injury with an AIS head >2.

5,441 patients (57%) suffered from severe TBI, and 2,800 (51%) from isolated severe TBI (Table 1). Overall, 14% (n = 779) of patients with severe TBI died. For patients who died, the median ISS was higher (median 26 vs. 20) and the GCS lower than for those who survived. Most patients died within the first 30 to 50 days after trauma (S1 Fig). Of the 1,546 patients with GCS 3, 1,133 (73%) were intubated on scene (S4 Table).

### Secondary transfers to MTCs

26% of patients (n = 2,492), or an average of 7 patients per day, were secondarily transferred from a regional hospital to the MTC. Patients with direct admission to an MTC had higher mortality (14%) than those with secondary transfer (8%) (Table 1).

The percentages of secondary transfer varied by injury type and age group (Table 2). Patients with isolated severe TBI and those aged ≥65 were more often first treated in a regional hospital and afterwards transferred to an MTC. On average, every third patient with an isolated severe TBI (34%) had first been admitted to a regional hospital and then transferred to an MTC (Table 2).

**Table 2:** Patients with and without traumatic brain injury (TBI) by age group and admission type.

	Direct admission			2° Transfer		
	Survived	Died	Total	Survived	Died	Total
**No TBI**	**2,824**	**346 (10.9%)**	**3,170**	**915**	**69 (7.0%)**	**984 (23.7%)**^**2**^
**Age group**						
16–24	327	18 (5.22)	345	72	0 (0.0)	72 (17.3)
25–34	397	29 (6.81)	426	88	1 (1.12)	89 (17.3)
35–44	363	23 (5.96)	386	85	1 (1.16)	86 (18.2)
45–54	527	19 (3.48)	546	144	4 (2.70)	148 (21.3)
55–64	473	39 (7.62)	512	165	11 (6.25)	176 (25.6)
65–74	333	50 (13.1)	383	154	9 (5.52)	163 (29.9)
75–84	297	87 (22.7)	384	142	22 (13.4)	164 (29.9)
≥85	107	81 (43.1)	188	65	21 (24.4)	86 (31.4)
**TBI**^**1**^	**3,288**	**645 (16.4%)**	**3,933**	**1,374**	**134 (8.9%)**	**1,508 (27.7%)**^**2**^
**Age group**						
16–24	310	32 (9.36)	342	84	1 (1.18)	85 (19.9)
25–34	325	28 (7.93)	353	87	2 (2.25)	89 (20.1)
35–44	283	28 (9.00)	311	85	1 (1.16)	86 (21.7)
45–54	396	53 (11.8)	449	134	8 (5.63)	142 (24.0)
55–64	440	66 (13.0)	506	198	6 (2.94)	204 (28.7)
65–74	502	118 (19.0)	620	250	23 (8.42)	273 (30.6)
75–84	576	168 (22.6)	744	352	59 (14.4)	411 (35.6)
≥85	456	152 (25.0)	608	184	34 (15.6)	218 (26.4)
**Isolated TBI**	**1,527**	**321 (17.4%)**	**1,848**	**865**	**87 (9.1%)**	**952 (34.0%)**^**2**^
**Age group**						
16–24	105	8 (7.08)	113	54	1 (1.82)	55 (32.7)
25–34	123	5 (3.91)	128	50	0 (0)	50 (28.1)
35–44	97	12 (11.0)	109	54	1 (1.82)	55 (33.5)
45–54	146	17 (10.4)	163	70	4 (5.41)	74 (31.2)
55–64	191	22 (10.3)	213	109	4 (3.54)	113 (34.7)
65–74	239	64 (21.1)	303	164	19 (10.4)	183 (37.7)
75–84	338	102 (23.2)	440	232	39 (14.4)	271 (38.1)
≥85	288	91 (24.0)	379	132	19 (12.6)	151 (28.5)

1) All TBI patients, including patients with concomitant TBI and patients with isolated TBI.

2) Percentage of patients with secondary transfer to a major trauma center (MTC).

**Fig 2:**
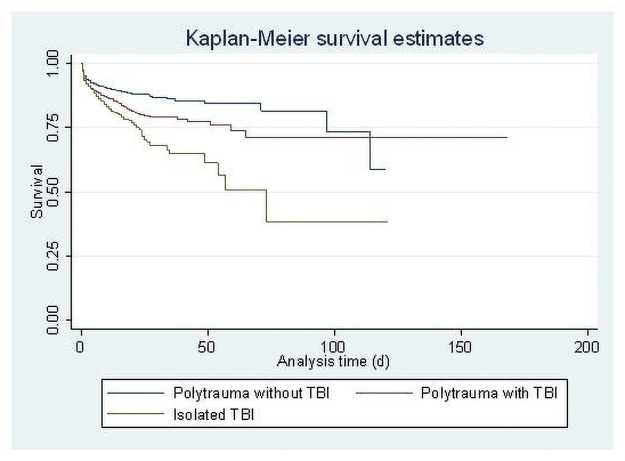
Mortality for patients without, with concomittant and with isolated TBI.

Mortality increased with increasing age—for both patients with direct admission and for those with secondary transfer (Table 2). In patients with direct admission to an MTC, mortality was lowest for those without TBI (11%), increased for those with concomitant TBI (16%) and highest for patients with isolated severe TBI (17%) (Table 2 and Fig 2). In patients with secondary transfer, there were only small differences in mortality between the three injury types (Table 2).

### Patients with GCS 13–15

Two thirds of patients (69%, n = 6,599) had a favourable GCS between 13–15 on admission to the ED (Table 1, S1 Table). Among patients with high GCS, about 40% of young patients and between 60%-75% of elderly (>65years) patients nevertheless suffered from severe TBI (S2 and S3 Tables).

### Cox proportional hazard models

Crude analysis showed a significantly elevated hazard for death of 1.63 (95% CI 1.40–1.89) for direct admission to the MTC compared to secondary transfer. Crude analysis showed a significantly elevated hazard for death of 1.48 (95% CI 1.28–1.70) for concomitant severe TBI and a hazard ratio (HR) of 1.82 (95% CI 1.58–2.09) for isolated severe TBI, compared to polytrauma patients without TBI.

In the analyses including age group, GCS, ISS, MTC, transfer type and gender, results were less severe for patients with concomitant severe TBI; the results of the latter patients were comparable results to those of patients with polytrauma without TBI. Patients with isolated severe TBI have a persistent higher mortality hazard (HR 1.67, 95%CI 1.44–1.95). Furthermore, mortality was higher with each point increase in ISS (HR 1.03, 95% CI 1.03–1.04), with greater age ≥55 years (with age 45 as the baseline) and for patients with primary admission to an MTC (HR 1.66, 95% CI 1.42–1.94). No significant difference was found for females compared to males (HR 0.96, 95% CI 0.85–1.08). The hazard for death was significantly lower for patients with higher GCS categories (Table 3).

**Table 3:** HR for death from adjusted Cox proportional hazard models.

	HR (95% CI)	p
**Injury type**		
Polytrauma^1^	Baseline	
TBI^2^	0.91 (0.79–1.05)	0.189
Isolated TBI^3^	1.67 (1.44–1.95)	<0.001
**Gender**		
Male	Baseline	
Female	0.96 (0.85–1.08)	0.481
**GCS**		
3	Baseline	
4–5	0.82 (0.58–1.16)	0.263
6–8	0.41 (0.32–0.54)	<0.001
9–12	0.28 (0.23–0.34)	<0.001
13–15	0.08 (0.06–0.09)	<0.001
missing	0.45 (0.34–0.59)	<0.001
**ISS**	1.03 (1.03–1.04)	<0.001
**Age group**		
16–24	0.72 (0.51–1.03)	0.069
25–34	0.80 (0.57–1.11)	0.179
35–44	0.81 (0.57–1.14)	0.231
45–54	Baseline	
55–64	1.50 (1.13–1.98)	0.005
65–74	2.21 (1.71–2.86)	<0.001
75–84	3.97 (3.11–5.06)	<0.001
≥85	7.51 (5.83–9.68)	<0.001
**Admission type**		
1° admission	1.66 (1.42–1.94)	<0.001
2° transfer	Baseline	

Cox proportional hazards models adjusted for gender, age, Glasgow Coma Scale Score (GCS), Injury Severity Score (ISS) and Major Trauma Center (MTC). 1) Patients with polytrauma (ISS>15) and without TBI. 2) Patients with polytrauma and concomitant TBI. 3) Patients with isolated TBI.

### Testing for interaction

Clear evidence for interaction between injury type and transfer type was present (p = 0.009) and with age group (p<0.001). No interaction was found between injury type and gender (p = 0.345). Separate Cox regressions by injury type showed that the mortality hazard for patients with direct admission to the MTC remained significantly higher than for patients with secondary transfer, for all injury types. Mortality HRs were unaffected by differences in gender, GCS category or ISS. However, polytrauma patients without TBI exhibited higher HRs with increasing age, especially above the age of 75 years (Table 4).

**Table 4:** HR for death from separate Cox proportional hazards models by injury type.

	No TBI	TBI	Isolated TBI
**Direct admission** **(yes vs no)**	1.48 (1.13–1.94)	1.41 (1.02–1.95)	2.11 (1.64–2.70)
**Female vs male patient**	1.04 (0.84–1.28)	0.96 (0.76–1.20)	0.88 (0.71–1.08)
**GCS**			
**3**	Reference	Reference	Reference
**4–5**	0.65 (0.36–1.17)	0.43 (0.16–1.18)	1.26 (0.75–2.09)
**6–8**	0.50 (0.32–0.79)	0.51 (0.31–0.83)	0.29 (0.19–0.46)
**9–12**	0.30 (0.21–0.43)	0.29 (0.20–0.42)	0.26 (0.19–0.35)
**13–15**	0.08 (0.06–0.10)	0.08 (0.06–0.11)	0.07 (0.05–0.09)
**missing**	0.56 (0.36–0.87)	0.64 (0.39–1.07)	0.29 (0.17–0.51)
**Age group**			
**16**	0.96 (0.52–1.78)	0.70 (0.42–1.16)	0.53 (0.24–1.17)
**25**	1.38 (0.80–2.38)	0.73 (0.44–1.21)	0.29 (0.11–0.78)
**35**	1.24 (0.69–2.21)	0.60 (0.33–1.07)	0.78 (0.38–1.59)
**45**	Reference	Reference	Reference
**55**	2.34 (1.42–3.84)	1.56 (1.01–2.40)	0.91 (0.51–1.64)
**65**	2.72 (1.66–4.46)	1.76 (1.17–2.64)	2.37 (1.45–3.87)
**75**	5.92 (3.75–9.36)	3.57 (2.41–5.27)	3.28 (2.05–5.24)
**≥85**	15.5 (9.66–24.7)	7.86 (5.20–11.8)	4.83 (2.95–7.89)
**ISS**	1.02 (1.02–1.03)	1.05 (1.04–1.05)	1.03 (1.03–1.04)

Derived Cox proportional hazards models adjusted for gender, age, Glasgow Coma Scale Score (GCS), Injury Severity Score (ISS) and Major Trauma Center (MTC), including interaction parameters for direct admission (yes vs no) by injury type and age group.

## Discussion

### Summary of results

About every fourth patient treated at one of the 12 MTCs in Switzerland with polytrauma and/or severe traumatic brain injury was first admitted to a regional hospital before being transferred to the MTC. We found that elderly patients and those with isolated traumatic brain injury were more likely to be secondarily transferred than were younger patients. Two thirds of patients were classified with a GCS 13–15, but 40% of young patients with this high GCS and the surprisingly high value of 60%-75% of the elderly had a severe TBI as diagnosed by the MTC. Cox regression models revealed a greater hazard for death for patients with direct admission to the MTC, for all injury types. Polytrauma patients without severe TBI had a lower mortality than patients with concomitant or isolated severe TBI.

### Strengths and weaknesses

We present one of the first nationwide analysis using patients from the Swiss Trauma Register. Although this is a registry based, retrospective study for a limited time period, we analyzed a large sample of standardized collected data of Swiss Trauma patients. Accordingly, 95% confidence intervals are narrow. STR is a reliable data set, which includes full prehospital data. To account for interaction between injury type and transfer type and between injury type and age group, we present data stratified by injury type. To avoid a clustering effect of MTCs, these were included in the adjusted Cox model.

### Comparison with literature

#### Effect of age

In our analysis, age ≥55 years was associated with an increased hazard for death in polytrauma patients with and without concomitant TBI and age ≥65 years was associated with an increased hazard for death in patients with isolated TBI. In 2014, Newgard et al. published revised triage criteria for older adults (≥55 years), taking into account the different physiology and higher mortality in these patients [17]. A similar effect of age, starting at around ≥60 years, had also been found in different analyses using patients from the UK trauma registry TARN, the Japanese national trauma data base, French trauma registry and the German trauma registry (RISC and RISC2 score) [30–35]. In a Swedish study at a cut-off at ≥60 years, patients with isolated severe TBI of AIS<2, were observed to have longer times from admission to the first CT scan and were less often triaged to the highest priority level, despite similar AIS scores to younger patients [36].

#### Triage of elderly and TBI patients

In a statewide analysis in Maryland (U.S.), Chang et al. found that patients aged ≥65 years were significantly more often undertriaged than younger patients. They defined major trauma patients with need for MTC as those who met the criteria of the American College of Surgeons, and were declared to be first priority by the ambulance team—critically ill or injured patients requiring immediate attention. Our study showed that the percentage of secondary transfer to an MTC increases with increasing age, starting at 45 years, but excluding the oldest age group of ≥85 years. Furthermore, we found that patients with isolated TBI were more often secondarily transferred than polytrauma patients without or with concomitant TBI. This finding is new.

Additionally, we found that 40% of the young, 60% of patients aged ≥65 years and even 76% of those ≥85 years had a high GCS of 13–15 on arrival to the ED, although they were suffering from severe TBI. This shows that the GCS, while showing good correlation with overall mortality, is not discriminatory for triage of TBI patients. For this reason, Van Rein et al. recommended in their review that serial GCS scores could be helpful for the evaluation of suspected TBI in the context of TBI [1].

#### Amount of “undertriage”

The Center for Disease Control and the American College of Surgeon recommend aiming for an undertriage rate of less than 5%, while the overtriage rate should not exceed 25–35% [10, 25]. We did not determine the amount of overtriage in this study, but found that 26% of patients treated at the MTCs in Switzerland had been admitted to a regional hospital before. This corresponds to an undertriage above the 5% suggested by the American College of Surgeons. One reason for this might be that in Switzerland no standardized triage criteria are used by the different prehospital care providers. Another explanation could be that, it can be especially challenging to determine the GCS of elderly patients and therefore, undertriage might be more frequent. Several published studies have reported that older patients are more often initially undertriaged [12, 14–16]. One potential problem with determining the amount of undertriage could be the partial coverage of all injured in our study. We only analysed patients with primary or secondary referral to the MTC. What remains unclear is how many patients with TBI (AIS>2) remain in peripheral hospitals. Therefore, the actual amount of undertriage might even be higher than the 26% we found in our analysis.

#### Mortality

There is no evidence as to whether undertriage and subsequent inter-hospital transfer leads to higher mortality or whether this “extra way” has little or no impact on mortality [3, 9, 19, 20–22]. It was interesting that, in our study, patients directly admitted to the MTC had a higher mortality than those with secondary transfer. This result from crude models was robust to adjustment for covariates and also to separate analysis by injury type. It is probable that obviously severely injured patients are more easily recognized on scene and therefore directly admitted to an MTC. On the other hand, patients with high initial GCS or with less obvious injuries are first brought to a regional hospital.

## Conclusions

Our analyses showed that patients with isolated severe TBI and elderly patients were more often secondarily transferred to the MTC. Furthermore, the majority of patients, especially in the elderly population, presented with favourable initial GCS values. Therefore, careful triage in the field is important—especially in the elderly and in patients with TBI. Furthermore, severe TBI is still possible in patients with high GCS values. In case of doubt, triage to an MTC is preferable. As secondary referrals are high and to prevent potential overloading of MTCs, a better standardized triage scheme could possibly help to lower the inter-hospital transfer rates.

## Supporting information

S1 Fig Mortality for all patients. (TIF)

S1 Table GCS on admission to the emergency department in patients with TBI. (DOCX)

S2 Table Age group and injury type in patients with GCS 13–15 on admission to the emergency department. (DOCX)

S3 Table Severe TBI with initial GCS 13–15. (DOCX)

S4 Table Glasgow Coma Scale and prehospital intubation status by survival. (DOCX)

S1 Appendix (DOCX)
