# Supplementary figures and images for: Inter-hospital transfer of polytrauma and severe traumatic brain injury patients: Retrospective nationwide cohort study using data from the Swiss Trauma Register
Source: PLoS One. 2021 Jun 18;16(6):e0253504. doi: 10.1371/journal.pone.0253504 (PMC8213144; doi:10.1371/journal.pone.0253504)

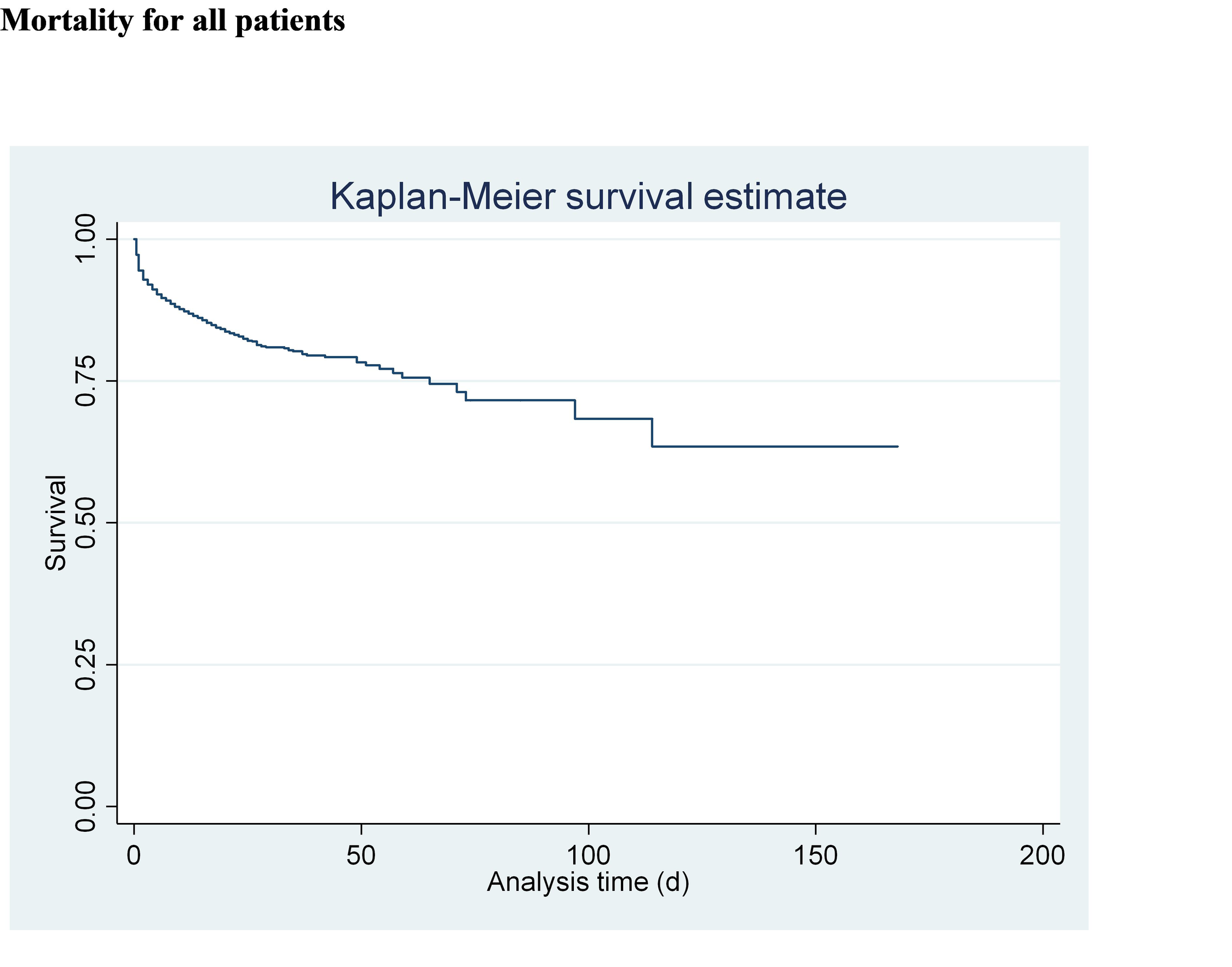

Supplement: S1 Fig — (TIF) [file pone.0253504.s001.tif]
