# Supplementary material for: Inter-hospital transfer of polytrauma and severe traumatic brain injury patients: Retrospective nationwide cohort study using data from the Swiss Trauma Register
Source: PLoS One. 2021 Jun 18;16(6):e0253504. doi: 10.1371/journal.pone.0253504 (PMC8213144; doi:10.1371/journal.pone.0253504)
Supplement: S1 Table — (DOCX) [file pone.0253504.s002.docx]

**S1 Table. GCS on admission to the emergency department**

| **GCS** | **3** | **4-5** | **6-8** | **9-12** | **13-15** |
| --- | --- | --- | --- | --- | --- |
| No TBI | 578 (14.5) | 26 (0.65) | 77 (1.93) | 234 (5.85) | 3,084 (77.1) |
| TBI | 618 (24.7) | 20 (0.80) | 98 (3.92) | 244 (9.75) | 1,522 (60.8) |
| Isolated TBI | 350 (12.9) | 27 (1.00) | 84 (3.09) | 262 (9.65) | 1,993 (73.4) |
