# Supplementary material for: Inter-hospital transfer of polytrauma and severe traumatic brain injury patients: Retrospective nationwide cohort study using data from the Swiss Trauma Register
Source: PLoS One. 2021 Jun 18;16(6):e0253504. doi: 10.1371/journal.pone.0253504 (PMC8213144; doi:10.1371/journal.pone.0253504)
Supplement: S2 Table — (DOCX) [file pone.0253504.s003.docx]

**S2 Table. Age group and injury type in patients with GCS 13-15 on admission to the emergency department**

| **Age group** | **No TBI** | **TBI** | **Isolated TBI** | **All TBI^1^** |
| --- | --- | --- | --- | --- |
| 16-24 | 303 (56.6) | 123 (23.0) | 109 (20.4) | 232 (43.4) |
| 25-34 | 368 (58.9) | 128 (20.5) | 129 (20.6) | 257 (41.1) |
| 35-44 | 350 (58.0) | 138 (22.9) | 115 (19.1) | 253 (42.0) |
| 45-54 | 539 (59.8) | 202 (22.4) | 160 (17.8) | 362 (40.2) |
| 55-64 | 546 (53.7) | 231 (22.7) | 239 (23.5) | 470 (46.3) |
| 65-74 | 410 (41.9) | 221 (22.6) | 347 (35.5) | 568 (58.1) |
| 75-84 | 385 (32.9) | 282 (24.1) | 503 (43.0) | 785 (67.1) |
| ≥85 | 183 (23.7) | 197 (25.6) | 391 (50.7) | 588 (76.3) |

1. All TBI patients, including patients with concomitant TBI and patients with isolated TBI.
