# Supplementary material for: Inter-hospital transfer of polytrauma and severe traumatic brain injury patients: Retrospective nationwide cohort study using data from the Swiss Trauma Register
Source: PLoS One. 2021 Jun 18;16(6):e0253504. doi: 10.1371/journal.pone.0253504 (PMC8213144; doi:10.1371/journal.pone.0253504)
Supplement: S3 Table — (DOCX) [file pone.0253504.s004.docx]

**S3 Table. Severe TBI with initial GCS 13-15**

|  | **Direct admission** | | | **2° Transfer** | | |
| --- | --- | --- | --- | --- | --- | --- |
|  | **Survived** | **Died** | **Total** | **Survived** | **Died** | **Total** |
| **No TBI**  **Age group**  16-24  25-34  35-44  45-54  55-64  65-74  75-84  ≥85 | **2,234**  243  288  276  426  396  280  235  90 | **65 (2.8%)**  1 (0.41)  3 (1.04)  3 (1.09)  2 (0.47)  4 (1.01)  7 (2.50)  19 (8.09)  26 (28.9) | **2,299**  244  291  279  428  400  287  254  116 | **761**  59  77  71  110  146  121  119  58 | **24 (3.1%)**  0 (0)  0 (0)  0 (0)  1 (0.91)  0 (0)  2 (1.65)  12 (10.1)  9 (15.5) | **785**  59  77  71  111  146  123  131  67 |
| **TBI***  **Age group**  16-24  25-34  35-44  45-54  55-64  65-74  75-84  ≥85 | **2,267**  161  188  183  253  313  351  455  363 | **100 (4.2%)**  1 (0.62)  1 (0.53)  1 (0.55)  2 (0.79)  2 (0.64)  15 (4.27)  28 (6.15)  50 (13.8) | **2,367**  162  189  184  255  315  366  483  413 | **1,107**  70  68  69  107  154  198  282  159 | **41 (3.6%)**  0 (0)  0 (0)  0 (0)  0 (0)  1 (0.65)  4 (2.02)  20 (7.09)  16 (10.1) | **1,148**  70  68  69  107  155  202  302  175 |
| **Isolated TBI**  **Age group**  16-24  25-34  35-44  45-54  55-64  65-74  75-84  ≥85 | **1,183**  63  85  69  104  151  195  282  234 | **68 (5.4%)**  1 (1.59)  1 (1.18)  0 (0)  1 (0.96)  1 (0.66)  11 (5.64)  19 (6.74)  34 (14.53) | **1,251**   \| 64  86  69  105  152  206  301  268 \| \| --- \| | **712**  45  43  46  55  86  137  186  114 | **30 (4.0%)**  0 (0)  0 (0)  0 (0)  0 (0)  1 (1.16)  4 (2.92)  16 (8.60)  9 (7.89) | **742**  45  43  46  55  87  141  202  123 |

*All TBI patients, including patients with concomitant TBI and patients with isolated TBI.
