# Supplementary material for: Inter-hospital transfer of polytrauma and severe traumatic brain injury patients: Retrospective nationwide cohort study using data from the Swiss Trauma Register
Source: PLoS One. 2021 Jun 18;16(6):e0253504. doi: 10.1371/journal.pone.0253504 (PMC8213144; doi:10.1371/journal.pone.0253504)
Supplement: S4 Table — (DOCX) [file pone.0253504.s005.docx]

**S4 Table. Glasgow Coma Scale and prehospital intubation status by survival**

| **Variable** | **Survived** | | | **Died** | | | **Total** |
| --- | --- | --- | --- | --- | --- | --- | --- |
| **Prehospital intubation** | yes | no | missing | yes | no | missing |  |
| **Glasgow Coma Scale**  3  4-5  6-8  9-12  13-15  **m**issing | 642  9  22  35  140  147 | 133  28  157  483  5,088  121 | 111  2  17  84  1,141  41 | 491  6  8  0  8  52 | 109  24  47  114  171  15 | 60  4  8  24  51  2 | 1,546  73  259  740  6,599  378 |
