# Supplementary material for: Inter-hospital transfer of polytrauma and severe traumatic brain injury patients: Retrospective nationwide cohort study using data from the Swiss Trauma Register
Source: PLoS One. 2021 Jun 18;16(6):e0253504. doi: 10.1371/journal.pone.0253504 (PMC8213144; doi:10.1371/journal.pone.0253504)
Supplement: S1 Appendix — (DOCX) [file pone.0253504.s006.docx]

**S1 Appendix**

**AIS head codes >2 according to the STR definitions**

110404.3

110606.3

110806.3

110808.3

113000.6

116000.3

116002.3

116004.5

120199.3

120202.5

120204.3

120205.4

120206.3

120299.3

120402.5

120404.5

120406.5

120499.4

120602.4

120603.4

120802.4

120804.5

120806.3

120899.3

121002.5

121003.6

121004.4

121005.5

121006.3

121099.3

121402.5

121404.4

121405.5

121406.3

121499.3

121602.4

121604.3

121606.3

121699.3

121802.5

121804.3

121805.4

121806.3

121899.3

122002.4

122003.5

122004.5

122005.6

122006.4

122007.5

122099.4

122202.4

122204.3

122299.3

122399.3

122402.4

122404.5

122406.4

122407.4

122408.5

122499.3

122502.4

122504.3

122599.3

122602.4

122603.5

122604.5

122605.6

122606.4

122607.6

122608.5

122699.3

122702.4

122704.3

122799.3

122802.5

122803.6

122804.3

122805.4

122806.3

122899.3

123002.4

123003.5

123004.5

123099.4

131605.3

131806.3

140202.5

140204.5

140208.5

140210.5

140212.6

140214.6

140216.6

140218.6

140299.5

140402.3

140403.3

140404.4

140405.5

140410.3

140414.3

140418.4

140422.5

140426.3

140430.4

140434.5

140438.3

140442.4

140446.5

140450.3

140458.3

140462.3

140472.4

140473.3

140474.3

140476.5

140477.3

140478.3

140499.3

140602.3

140604.3

140606.3

140608.4

140610.5

140611.3

140612.3

140614.3

140616.4

140618.5

140620.3

140622.3

140624.4

140625.4

140626.5

140627.5

140628.4

140629.3

140630.3

140632.4

140634.5

140636.5

140638.3

140640.4

140641.5

140645.4

140646.5

140647.3

140648.5

140649.4

140650.3

140651.3

140652.4

140654.4

140655.5

140656.5

140660.3

140662.3

140664.4

140666.5

140668.3

140670.3

140672.4

140674.5

140676.3

140677.4

140680.3

140681.3

140682.3

140683.5

140686.4

140687.3

140688.3

140690.3

140691.3

140692.5

140695.3

140698.3

140699.3

140799.3

150200.3

150202.3

150204.3

150206.4

150404.3

150406.4

150408.4

161006.3

161007.4

161008.4

161011.5

161012.5

161013.5
